# Measuring Protons with Photons: A Hand-Held, Spectrophotometric pH Analyzer for Ocean Acidification Research, Community Science and Education

**DOI:** 10.3390/s22207924

**Published:** 2022-10-18

**Authors:** William Pardis, Kalina C. Grabb, Michael D. DeGrandpre, Reggie Spaulding, James Beck, Jonathan A. Pfeifer, David M. Long

**Affiliations:** 1 Flathead Valley Community College, Kalispell, MT 59901, USA; 2 Woods Hole Oceanographic Institution, Applied Ocean Physics and Engineering, Woods Hole, MA 02543, USA; 3 Massachusetts Institute of Technology/Woods Hole Oceanographic Institution Joint Program in Oceanography/Applied Ocean Science & Engineering, Woods Hole, MA 02543, USA; 4 Sunburst Sensors LLC, Missoula, MT 59802, USA; 5 Department of Chemistry and Biochemistry, College of Humanities and Sciences, University of Montana, Missoula, MT 59812, USA; 6 Department of Chemistry and Biochemistry, College of Science and Mathematics, California Polytechnic State University, San Louis Obispo, CA 93407, USA

**Keywords:** chemical sensor, coastal ocean monitoring, science education, colorimetric pH measurement, meta-cresol purple

## Abstract

Ocean Acidification (OA) is negatively affecting the physiological processes of marine organisms, altering biogeochemical cycles, and changing chemical equilibria throughout the world’s oceans. It is difficult to measure pH broadly, in large part because accurate pH measurement technology is expensive, bulky, and requires technical training. Here, we present the development and evaluation of a hand-held, affordable, field-durable, and easy-to-use pH instrument, named the pHyter, which is controlled through a smartphone app. We determine the accuracy of pH measurements using the pHyter by comparison with benchtop spectrophotometric seawater pH measurements, measurement of a certified pH standard, and comparison with a proven in situ instrument, the iSAMI-pH. These results show a pHyter pH measurement accuracy of ±0.046 pH or better, which is on par with interlaboratory seawater pH measurement comparison experiments. We also demonstrate the pHyter’s ability to conduct both temporal and spatial studies of coastal ecosystems by presenting data from a coral reef and a bay, in which the pHyter was used from a kayak. These studies showcase the instrument’s portability, applicability, and potential to be used for community science, STEM education, and outreach, with the goal of empowering people around the world to measure pH in their own backyards.

## 1. Introduction

As atmospheric carbon dioxide (CO_2_) levels continue to increase from 280 parts per million volume (ppmv) in preindustrial times to 420 ppmv in 2022 [1], oceans have played a significant role in moderating CO_2_ concentrations through atmospheric uptake. The ocean sink accounts for nearly a third of anthropogenic carbon emitted to the atmosphere; however, this is not without consequences [2]. Introducing CO_2_ into the ocean produces carbonic acid, reducing ocean pH, a process that is popularly termed ocean acidification (OA) [3]. pH is defined as:

pH = −log (a _(H+)_) (1)

where a_H+_ is the activity of the hydrogen ion (H^+^) [4]. As a controlling variable in aqueous chemical equilibria, even small changes in pH can alter the chemical composition of natural water, potentially affecting essential physiological processes (e.g., enzymes, cell equilibria, and gene expression) [5,6,7]. Within ocean ecosystems, the current reduction in pH has significant implications for continued carbon sequestration and calcium carbonate saturation [3], the latter of which is used as a proxy for calcifying organisms’ ability to produce shells. In addition to the direct connection with OA, pH is also a proxy in natural aquatic systems due to its relationship with biological productivity through the inorganic carbon cycle (i.e., respiration and photosynthesis) [8]. Understanding and monitoring these changes within the environment is crucial for studies of, for example, climate, marine ecosystem health, and fisheries [9,10].

Global impacts of OA and implications for the overall carbon budget remain poorly constrained, largely due to the lack of monitoring on appropriate spatial and temporal scales. In particular, coastal ecosystems (e.g., salt marshes, estuaries, seagrass beds, coral reefs, and mangroves), which occupy a small fraction of the world’s oceans, can disproportionately influence the global carbon budget due to high productivity and rapid carbon cycling. Widespread pH measurements are particularly important to assess the natural variations within coastal marine ecosystems, where some of the most extreme pH variability occurs [6,11]. In coastal regions, pH can fluctuate upwards of one pH unit due to biological activity alone, yet anthropogenic influences, such as sewage or industrial discharge, also often compound the coastal variations in pH [6,11,12,13,14]. Since pH can vary widely within local regions, it is difficult to monitor, understand, and interpret these changes without the widespread availability of pH measurements. 

Infrequent monitoring on relevant spatial and temporal timescales is due in large part to the difficulty in making accurate pH measurements, the need for technical training, and the requirement for expensive instrumentation for the most accurate measurements. A wide array of methods and technologies have been developed to make pH measurements, including a variety of electrochemical sensors [7,15,16], spectrophotometric laboratory methods [17,18,19,20,21,22], and spectrophotometric in-situ methods [23,24]. Electrochemical sensors include the traditional glass electrode and the more recently developed extended gate field effect transistor (EGFET) and ion-sensitive field effect transistor (ISFET) [7,16,25]. Spectrophotometric pH analysis is preferred for seawater measurements due to the method’s long-term stability and high precision and accuracy, providing calibration-free performance relative to electrochemical methods [17,24,26,27]. Specifically, the sulfonephthalein indicator m-cresol purple (mCP), with a pK_a_ of ~8.0 at seawater salinity, is the optimal indicator for marine environments [17,26,28]. Laboratory spectrophotometric methods with mCP led to the development of in situ analyzers, such as the Submersible Autonomous Moored Instruments (SAMI), which make stable and precise pH measurements when deployed in marine environments [23,24]. Of these pH measurement methods, none are both high-performance and affordable; most require trained scientific personnel to operate, deploy, maintain, and recover the instruments. A simple, low-cost, handheld spectrophotometric pH device that can accurately (0.01–0.02 pH units) measure pH would fill an important niche for OA studies. One previously developed handheld pH instrument was created for a low cost with high accuracy (0.01 pH units), with the intention for users to “do-it-yourself (DIY)” to create the instrument [29]. While this instrument could potentially enable widespread pH measurements, the DIY method still requires time, resources, and skills for the user to be able to make the instrument and conduct pH measurements. Most scientists that are focused on OA will not have the time or resources to build DIY instruments, so ultimately, DIY devices have not been extensively used. Due to these limitations in currently available pH instruments, pH measurements remain inaccessible to many who are living on or near the coast (i.e., potential citizen scientists), where pH measurements frequently fluctuate and are most necessary and directly relevant to society. 

Here we describe the recent development of a handheld, low-cost, pH meter, called the pHyter. The pHyter design is based on mCP chemistry with the desired qualities (e.g., portable, durable, self-contained, and wireless operation) for field measurements of aquatic pH, but is specifically designed for efficient commercial manufacturing (i.e., minimal labor, mass-produced components, etc.). Analytical laboratory characterizations, as well as field deployments in North America and the South Pacific, were used to test and validate the pHyter. The pHyter prototype measured seawater pH at a higher level of accuracy than traditional electrochemical probes (±0.033 pH or better), costs a mere fraction of the price of autonomous spectrophotometric methods, and is field-durable and easy to use. While many instruments are moving towards automation, the pHyter purposefully requires human involvement–engaging people enables the pHyter to be a powerful tool for outreach, education, and community-based science. As presented in this study, the pHyter increases our ability to make high-quality, and geographically and temporally widespread pH measurements, particularly in remote coastal environments. 

## 2. Materials and Methods

### 2.1. Spectrophotometric pH Chemistry

#### 2.1.1. pH Chemistry

The pHyter uses the spectrophotometric pH method described previously [23,28]. Briefly, the method measures the equilibrium of the indicator meta–Cresol Purple (mCP) with hydrogen ion concentration in solution:

(2) $${\mathrm{HL}}^{-}\;⇌{\;H}^{+}+{\;L}^{2-}$$

where HL^−^ and L^2−^ are the first and second deprotonated forms of the mCP indicator, which have absorbance peaks at 434 nm and 578 nm, respectively. In seawater, the fully protonated form of mCP (H_2_L) is insignificant and, hence, the equilibrium is dominated by the second apparent acid dissociation constant. Seawater pH on the total hydrogen ion scale [30] can thus be calculated from the acid dissociation equilibrium expression (2) in combination with Beer’s Law:

(3) pHT=pKa′+log10R−e1e2−Re3

where pH_T_ is the total hydrogen ion concentration, the accepted standard for seawater pH measurements [17,30], and e_1_, e_2_, and e_3_ are ratios of the molar absorption coefficients associated with wavelengths of 578 and 434 nm [26].

(4) $$R=\frac{A_{578}}{A_{434}}$$

where A_λ_ is the absorbance at a wavelength λ. The absorption coefficients and their temperature dependence were determined experimentally for the pHyter and are embedded in the firmware to calculate the pH upon measurement. 

The acid dissociation coefficient (pK_a_’) of purified mCP (PmCP) has been previously determined [26,31,32]. For a salinity of 35, the pKa’ is calculated as follows:

(5) $${\mathrm{pK}}_{a}^{\prime }=-241.462+\frac{7085.72}{T}+43.8332*\ln\left(T\right)-0.0806406*T\;$$

where T is the temperature in Kelvin. The pHyter calculates this pK_a_’ based on the temperature measured at the bottom of the sample cuvette. Salinity is accounted for using equations derived by Mosley et al. for unpurified mCP [33]. Although Müller and Rehder [31] derived a more precise salinity dependence from salinity 0–40, their use of a combined pK_a_’·e_2_ term makes it impossible to account for the wide bandwidth of the pHyter’s light source. Therefore, combining Equation (5) with salinity dependence from Mosley et al., the temperature and salinity dependent equation used for the pHyter is:

(6) $${\mathrm{pK}}_{a}^{\prime }=\frac{7085.72}{T}+43.8332\;*\;\ln\left(T\right)-0.0806406\;*\;T-0.3238{\;*\;S}^{0.5}+0.0807\;*S-0.01157{\;*\;S}^{1.5}+0.000694{\;*\;S}^{2}-240.825\;$$

where S is salinity. At 298.15 K and salinity 35, pK’_a_ is 8.005. If impure dye is used, the impurity can be corrected for following Douglas and Byrne [34].

#### 2.1.2. Meta–Cresol Purple Dye

The use of meta-Cresol Purple (mCP) indicator in the pHyter is based on previous methods that have been reported in detail [17,19,26]. For this study, the indicator solution was purified by flash chromatography, following Liu et al. [35]. Purified mCP reagent was prepared at 8.2 × 10^−4^ mol·kg soln^−1^ in nanopure water. The indicator is adjusted to a pH of 8.0 to minimize perturbation of seawater samples, which is currently an average pH of 8.1 [1], but can vary from 7.4 to 8.6 [36]. Although used here to be consistent with spectrophotometric pH comparisons, a purified indicator would not be needed for most pHyter applications, because the error due to impurities is small compared to the pHyter accuracy. 

### 2.2. Instrument Design and Operation

#### 2.2.1. pHyter Components

**Figure 1:**
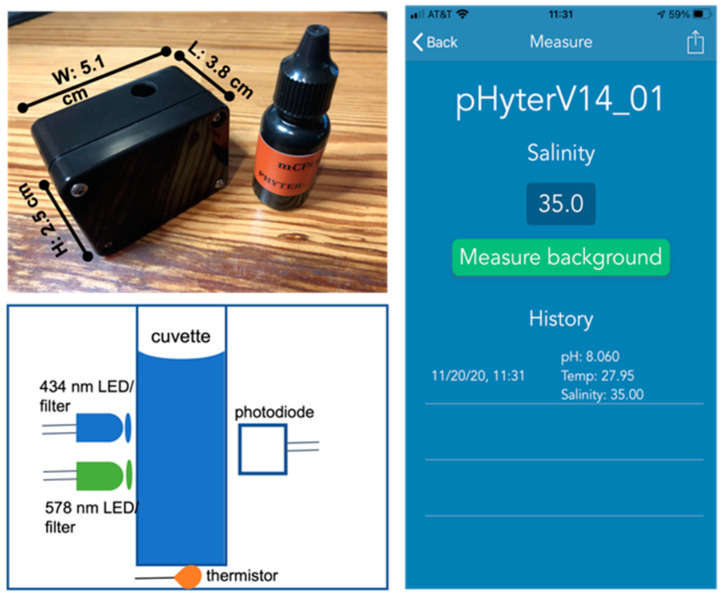
(The pHyter). The pHyter next to an mCP reagent bottle (**upper left**); pHyter optical design (**lower left**); and pHyter phone app (**right**).

The pHyter is a low-cost, two-wavelength colorimeter that fits in the palm of your hand (5.1 cm W × 3.8 cm L × 2.5 cm H, Figure 1) and weighs about 200 g. The instrument contains a 3-mL, 1-cm pathlength plastic cuvette, which is attached inside the housing, allowing for the addition of sample and reagent dye into the cuvette. A black rubber stopper is attached to the instrument to seal the cuvette (water- and light-tight) during measurement. Adjacent to the cuvette, optical bandpass filters (10 nm full width at half maximum (FWHM), Intor Inc., Socorro, NM, USA) are used to constrain LEDs to the peak absorbance wavelengths of the protonated and deprotonated dye. The optical intensity of each LED is measured with a single silicon photodiode with the LEDs turned on sequentially. The temperature within the optical cell is measured by a negative temperature coefficient (NTC) thermistor glued to the bottom of the cuvette. The pHyter is built around Arduino Atmel processors (ATmega328P), a low-power CMOS 8-bit microcontroller with 32K of program memory and 8K of data memory. The microprocessor sends data to HC-06, a class 2 slave Bluetooth module designed for transparent wireless serial communication. The HC-06, in turn, transmits data to a client application on a smartphone. The pHyter is powered by an internal nickel-cadmium battery (lifetime ~8 h) that can be wirelessly charged. 

#### 2.2.2. pHyter Operation

The pHyter uses Bluetooth low energy (BLE) to interface to an iPhone, referred to here as the user interface (UI), for the operation of the instrument. The UI also automatically stores raw light intensity, processed absorbance and pH data, time, temperature, and GPS location, and allows for the exporting of data in a spreadsheet-compatible file via typical social network sharing mechanisms. The UI can store 1000 or more samples and the data remains on the pHyter even when the instrument is disconnected or loses power.

With few materials and minimal training, pH can be measured on the pHyter using the following steps:

(1). Prior to each measurement, the user can input the salinity of the solution into the UI, otherwise, it will default to 35. Salinity readings can be obtained from a simple, inexpensive hand-held refractometer, e.g., a Brix Refractometer B01LW4HHRC, for ~$20.
(2). About 3 mL of sample solution is added to the sample cuvette, using a dropper or by submerging the pHyter directly in the water. Then the rubber stopper is placed on the sample cuvette and a blank (I_λ0_) is measured at both wavelengths by clicking the “Measure Background” button on the smartphone UI (Figure 1). After a few seconds, the UI will read “Measure pH”, noting that the background was successfully collected.
(3). The stopper is removed, and two drops of the indicator dye are added to the sample. The stopper is replaced, and the instrument is inverted 10 times slowly to mix the solution and dye. The transmitted intensity (I_λ_) of the sample with the dye addition is then measured at both wavelengths by pressing the “Measure pH” button on the UI.

At each wavelength, the absorbance is calculated by Equation (7):

(7) $$A=-\log_{10}\left(\frac{I_{\lambda }}{I_{\lambda 0}}\right)$$

The pH is calculated using the absorbance ratio, temperature, and input salinity (Equations (2)–(7)). 

### 2.3. Laboratory Evaluation

#### 2.3.1. pHyter Linearity

Absorbance linearity is important because it is an indication of absorbance accuracy, which affects pH accuracy. To test the linearity of the instrument, the absorbance of different concentrations of stock solutions of purified mCP (PmCP) in the singly-protonated (HL^−^) and deprotonated (L^2−^) forms were measured at 434 nm and 578 nm, respectively. A pH ~5 sodium acetate buffer and a pH ~11 NaOH solution were used to isolate the singly-protonated and deprotonated forms of the dye, respectively. Reagent stock solutions were diluted to achieve mCP concentrations ranging from 2.4 × 10^−6^ to 4.7 × 10^−5^ mol·kg soln^−1^ at pH 11 and 5.5 × 10^−6^ to 1.1 × 10^−4^ mol·kg soln^−1^ at pH 5, varying the absorbance from ~0.1 to 2 absorbance units. All solutions were adjusted to salinity 35 using NaCl. Measurements were completed in triplicate over the range of concentrations for all solutions. 

#### 2.3.2. Evaluation of Precision and Accuracy

To test the precision and accuracy of the pHyter, five pHyters were used to measure a Tris-buffered seawater solution obtained from the Scripps Institution of Oceanography (Tris batch T27). Measurements were made at temperatures between ~22 and 27 °C (ambient temperature), where the pH of the Tris solution ranges from 8.016 to 8.191. The Tris solution was measured on each pHyter at three different temperatures, with the pH of the Tris also calculated using equations in Müller et al. [37].

The pHyter’s accuracy and precision were further evaluated over a range of pH values through measurements by three pHyters and an ultraviolet-visible (UV-Vis) dual beam spectrophotometer (Varian Cary 100, referred to as the Cary spectrophotometer). Filtered seawater collected from the Pacific Northwest was amended with 0.1 N NaOH or 0.1 N HCl (certified standards, Fisher Scientific, Fair Lawn, NJ, USA) to obtain analyte solutions with varied pH. Eight measurements across four pH values (~7.25, 7.6, 7.9, and 8.3) were made using each pHyter and the Cary spectrophotometer. For measurements on the Cary spectrophotometer, a 10-cm cuvette was used to minimize the perturbation of seawater pH by the addition of PmCP [19]. The temperature of the cell was controlled using a water-jacketed cell holder, and samples were allowed to equilibrate to temperature for 10 min prior to blank measurements at 434 nm, 578 nm, and 740 nm (740 nm was used as a reference for light source drift since the indicators do not absorb light at this wavelength). Each pH analysis included three 80-µL additions of dye. After each addition, the dye was mixed with a micromagnetic stir bar, and absorbances at the three wavelengths were measured. pH was calculated using Equations (2)–(7). For these comparisons between the pHyter and the Cary spectrophotometer, the pH of the solution (pH_T_) was reported as the pH at zero dye concentration, which was extrapolated from the three dye additions [19,23]. 

### 2.4. Field Verification

#### 2.4.1. Field Measurements in Comparison to the iSAMI-pH

To verify the pHyter accuracy and precision for environmental pH measurements, the pHyter was compared to an in situ instrument, the iSAMI-pH (Sunburst Sensors, LLC, Missoula, MT, USA). The accuracy of the iSAMI-pH was verified at Sunburst to be better than ± 0.006 on Tris buffer at 25 °C and salinity 35 [37]. Measurements were made in a back reef environment in Tetiaroa, French Polynesia, in June of 2018. The iSAMI-pH was deployed for 36 h and collected pH measurements every 15 min. Measurements with the pHyter were taken in triplicate at regular intervals near the inlet of the iSAMI.

#### 2.4.2. Spatial Field Application

To test the mobility and versatility of the pHyter, the pHyter was used to map a spatial transect around a peninsula near Duxbury Bay, MA, USA, in August 2017. The pHyter was operated by one scientist from a one-person kayak and measurements were made in triplicate at each location by collecting water beside the kayak using a syringe.

## 3. Results and Discussion

### 3.1. Laboratory Evaluation

#### 3.1.1. pHyter Linearity

**Figure 2:**
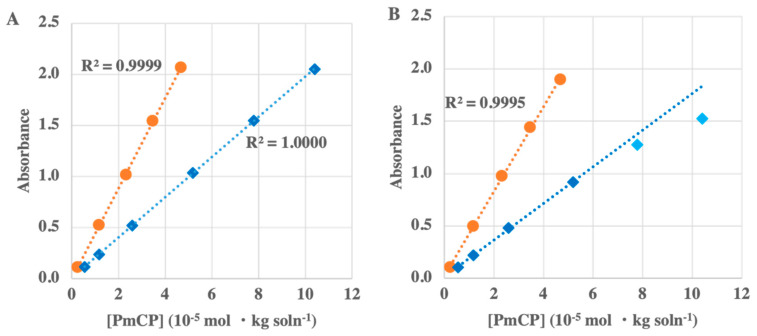
(Absorbance linearity). Absorbance was measured in triplicate by the pHyter over a range of mCP concentrations at pH 5 and 11. The orange circles represent 578 nm absorbance from the pH 11 solution; the blue diamonds represent 434 nm absorbance from the pH 5 solution. The light blue diamonds represent points deviating from the linear response at 434 nm. (**A**) A pHyter with 10-nm bandpass filters on each LED; (**B**) a pHyter without bandpass filters.

As discussed in Section 2.3.1, absorbance linearity with indicator concentration is critical for accurate pH measurements. The linearity tests indicate that the pHyter absorbances are linear from 0.1 to 2.0 absorbance units (AU) for both basic and acidic solutions when optical bandpass filters are used to narrow the LED output (Figure 2). However, the points start to fall off the regression line for acidic solution absorbances >1.5 when filters are not used. This suggests that there is significant out-of-bandpass light (stray light) at 434 nm that is removed by the bandpass filters. Optical filters increase the cost of producing a pHyter by ~$100. Therefore, the cost can be kept lower by omitting the filters and keeping absorbances below 1.5 AU at 434 nm to ensure that non-linearity does not affect pH accuracy. The absorbance range is readily controlled by the dropper used to add indicator to the sample in the pHyter cuvette, and absorbances are recorded in the pHyter measurement details on the app.

#### 3.1.2. pHyter Precision and Accuracy

The pHyter precision and accuracy were evaluated using measurements of Tris CRM. The pH of the certified Tris standard was measured at three different temperatures (determined by ambient temperature) on five different pHyters, and compared to the temperature-dependent certified Tris pH value [37]. These tests found an overall average offset between pHyter pH and certified pH of +0.026 ± 0.045 (+0.015 ± 0.011 when an apparent outlier is omitted, Table 1). Possible sources of offsets and variability could be errors in absorbance and/or temperature. The temperature is measured by a thermistor located at the bottom of the cuvette. This temperature does not exactly equal the sample temperature and differs significantly when the sample temperature and air temperature differ significantly. Since Tris pH changes by ~0.03 per degree Celsius, the accuracy of the pH measurement is highly dependent on an accurate temperature measurement. While benchtop instruments with experienced technicians can perform about 10 times better (±0.001) [38], this accuracy is comparable to an inter-laboratory comparison assessing the quality of seawater carbon dioxide measurements [39].

**Figure 3:**
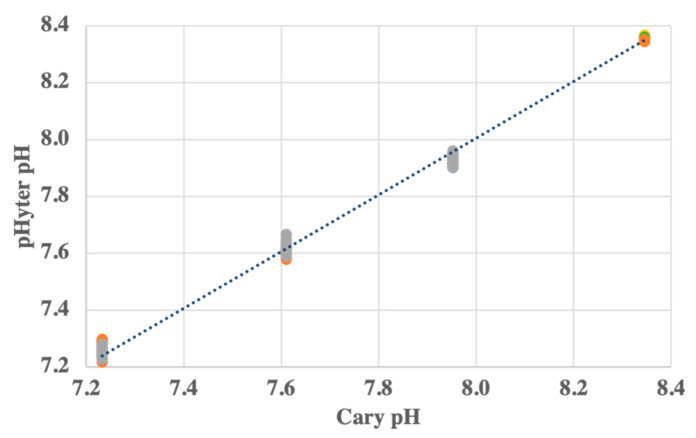
(pHyter versus Cary seawater pH measurements). Seawater pH measurements made by pHyters in relation to Cary UV-Vis measurements at four pH values between 7.2 and 8.3. The slope of the linear fit is 0.997, R^2^ = 0.998, n = 69). Six pHyters were used, shown in yellow, turquoise, green, blue, orange, and gray.

The pHyter was further tested by comparing seawater pH measurements between pH 7.2 and 8.3 using a Cary spectrophotometer and six pHyters (Figure 3). The pHyter was most accurate for the pH 7.9 and 8.3 seawater (+0.002 ± 0.011 and +0.001 ± 0.011, respectively), compared to the pH 7.2 and 7.6 seawater (+0.005 ± 0.022 and +0.010 ± 0.026, respectively). Each of these numbers reflects an average of 18–23 measurements. Although the pHyter is not as accurate or precise as the most reliable measurements on benchtop instruments, the accuracy of the pHyter is on par with other errors associated with benchtop spectrophotometric pH measurements, including inter-laboratory differences (±0.02) [39], dye impurities (±0.008) [40], and dye perturbation (±0.001–0.05) [19,23]. This is especially true for the pHyter measurements at typical seawater pH (7.9–8.3). The standard deviation at each pH reflects the deviation between instruments and falls within the range of interlaboratory comparisons of benchtop spectrophotometric pH measurements. These laboratory tests show that near typical sea surface pH, and operated by one user, the pHyter performs similarly to the widely accepted laboratory benchtop spectrophotometers in interlaboratory comparisons. It is yet to be determined how the pHyter will perform in interlaboratory comparisons, but given the simplicity of its use, we expect it to perform well.

### 3.2. Field Evaluation

#### 3.2.1. Temporal Field Measurements in Comparison to the iSAMI-pH

**Figure 4:**
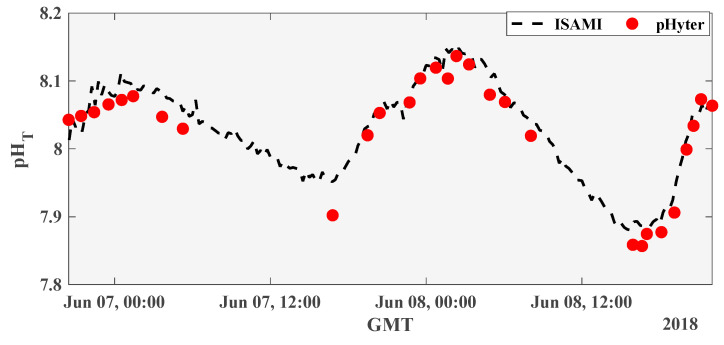
(pHyter and iSAMI pH measurements on a coral reef). pH measurements of back reef water in Tetiaroa, French Polynesia, over ~36 h. Data was collected continuously by the iSAMI (black dashed line) and periodically by the pHyter operator (red circles). The average difference between pHyter and iSAMI pH was −0.033 ± 0.066.

During measurements that were made simultaneously by the iSAMI-pH and the pHyter over 36 h on a reef in the South Pacific, the pHyter proved to measure similar pH trends as those observed by the iSAMI-pH, with an average difference between the pHyter and iSAMI of −0.033 ± 0.066 (Figure 4). The pHyter data are systematically lower than the iSAMI data, possibly due to errors in determining the absorbance constants for the pHyter, from dye perturbation of the seawater, or systematic temperature measurement errors. During these measurements, the air temperature was higher than the water temperature, causing the pHyter to measure temperature, on average, 0.7 °C higher than the iSAMI temperature. Seawater pH changes ~0.015 pH per degree Celsius, so the inaccuracy in the pHyter temperature measurement probably accounts for at least half of the pH offset between the pHyter and the iSAMI.

Through these tests, the pHyter also proved to be field durable and operational in a remote reef environment. The pH data obtained by both instruments reflects the diel variation in pH that would be expected as photosynthesis during the day consumes CO_2_, increasing pH, and respiration at night produces CO_2_, decreasing pH. Additionally, the measurements are made in real time, thus producing data instantly with no post-processing, allowing each measurement to immediately guide the user and influence the next sampling steps. Although it is unlikely the pHyter would be used to measure pH throughout the night, this time series demonstrates the pHyter’s ability to illuminate the temporal pH trends in coastal environments accurately, and in fine detail, similar to a much more sophisticated in situ instrument.

#### 3.2.2. Spatial Field Application

**Figure 5:**
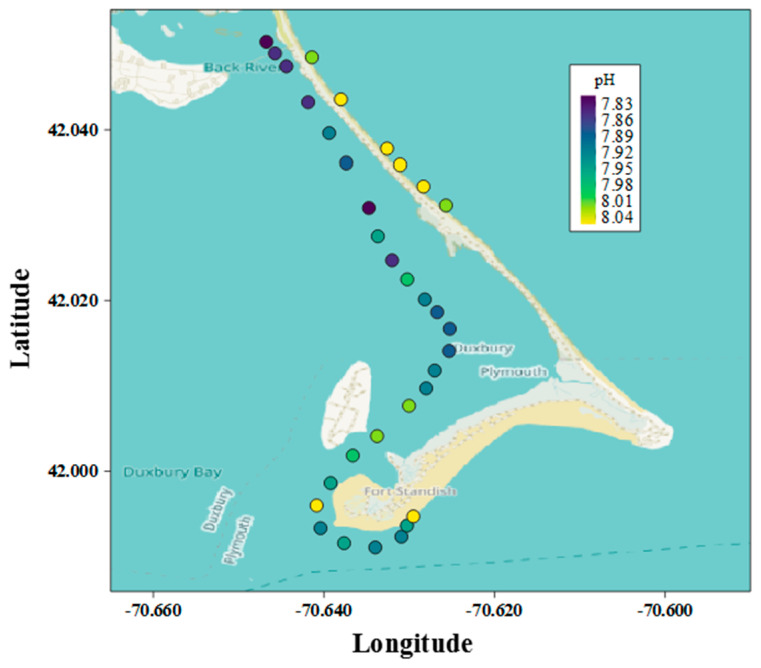
(pHyter pH measurements from a kayak). In situ surface water pH measurements made by the pHyter in Duxbury Bay, MA, are shown as circles at the measurement location. The circles are colored based on the pH measured (color scale in upper right corner). The pHyter was operated from a kayak over ~4 h.

pHyter measurements of pH across a spatial transect around a peninsula located in the Northeastern US near Duxbury Harbor, Massachusetts, demonstrate the ability of the pHyter to easily map spatial variations in pH (Figure 5). The pHyter was used in a single-person kayak, proving it to be easy to transport, field durable, and reliable, and demonstrating the feasibility of its use by citizen scientists. 

The pHyter measurements highlight the localized pH differences. Inside the bay, the pH increased along the transect from lower pH inland to higher pH towards the southern tip and open ocean (Figure 5). On the open-ocean side, the pH was consistent across the transect and higher than within the bay. Across this small distance (<5 miles) pH varied by nearly 0.2 pH units, which is a significant amount for many organisms (e.g., CaCO_3_ saturation) and biogeochemical processes [6,8,11]. Although some of this variation might be due to temporal and/or temperature differences (±1.9 °C) in the samples and pH change resulting from the natural diel cycle (the time difference between the first sample and the last sample was ~4 h, beginning at around 7:00 a.m.), the short time span of the study, as well as the trend of stable pH at the open-ocean sites, indicates that the difference is likely dominated by location. This pH range is also larger than the largest standard deviation of the laboratory seawater measurements completed by the pHyter (±0.026 at pH 7.61), validating the ability of the pHyter to resolve the pH differences within a coastal environment.

A small-scale survey such as this could inform local industries (e.g., aquaculture fisheries, seagrass remediation, or hatcheries) of favorable locations to optimize the placement of facilities. While these measurements would be possible to conduct using a pH electrode or in situ autonomous instrument, the electrode would probably be inaccurate, and in situ instrumentation would cost large amounts of money and require trained personnel.

### 3.3. The pHyter in Community-Based Science, Education, and Outreach

The pHyter provides an ideal opportunity to make pH measurements accessible and to empower people to monitor coastal and estuarine pH. The pHyter is designed to be a low-cost instrument that is easy to use for community-based science, education, and outreach. Through community-based science, the pHyter engages people in collecting scientific data and gives them a tool to identify, understand, and monitor small variations in pH in their local environment. As an example, the pHyter has been used in local communities in Washington State to monitor pH around the Olympic Peninsula. These observations can inform policy decisions and fuel grassroots support for issues that are currently threatening aquatic environments, including, for example, climate change, ocean acidification, eutrophication, and anthropogenic pollution. The pHyter is also an ideal educational tool as it lowers the price barrier to entry for students to collect their own data, ask and answer their own questions, and investigate local biogeochemical processes without sacrificing much accuracy. The pHyter has already been used in multiple education and outreach programs, empowering K-12, community college, and undergraduate students to make some of their first pH measurements in the ocean. Since the pHyter is designed to be used by nearly anyone who owns a smartphone, with minimal training, it requires very little technical skill. A trained and professional scientist completed all tests during this study; however, we have recently observed that after a one-hour training session, high school students were able to achieve accuracy and precision in pHyter measurements that are consistently higher than other pH methods. Clearly, the pHyter has the desirable attributes (e.g., affordable, easy-to-learn, accurate, handheld, and field-durable) to be used by communities, students, and the public to make pH measurements around the world. 

### 3.4. The Future of the pHyter

Sunburst Sensors has received a Phase 2 Small Business Innovation Research (SBIR) award from the National Oceanic and Atmospheric Administration (NOAA) to further develop the pHyter and to adapt it for other low-cost ocean monitoring applications for use in citizen science and education. We are currently collaborating with educators, community scientists, and non-profit organizations with outreach in the USA and the developing world. Future iterations of the pHyter will include the ability to upload data to a global database, accompanied by web-based teaching tools and a virtual user community, making OA studies and data sharing accessible to the global community. Additionally, we plan to develop similar instruments to measure total alkalinity, the partial pressure of CO_2_, and other parameters, such as dissolved O_2_ and fluorescent dissolved organic matter. There are challenges, including reducing manufacturing costs, providing technical support for a large number of users, and developing and maintaining apps for different smartphones. Despite these struggles, the goal is to produce a suite of instruments at a volume where people around the world can soon measure pH and other important seawater parameters and share data globally through a web portal. 

## 4. Conclusions

To expand spatial and temporal pH monitoring, a different approach is necessary from the traditional scientist-centric, “principal investigator” data collection model. There are communities around the world that live along coastlines that have invaluable local knowledge and are invested in their local environments, yet they do not currently have the tools to monitor their own waters. The pHyter, described here, was developed and validated for coastal pH measurements and meets the needs of community science. The cost of the parts is less than $200, it is small enough to be carried in a handbag or backpack, it is simple to use, and the accuracy of pH measurements with the pHyter is comparable to interlaboratory spectrophotometric pH measurements. The average pHyter accuracy and precision on Tris CRM measurements was +0.026 ± 0.045 pH. Comparison of benchtop spectrophotometric and pHyter measurements of seawater pH ranged from 0.001 to 0.010 difference. And finally, comparisons of in situ iSAMI and pHyter pH measurements on a reef in the South Pacific revealed a pH difference of −0.033 ± 0.066. The source of the error is not completely known but is probably related at least in part to temperature measurement error. This accuracy is a significant improvement over the current status quo pH measurement using an electrochemical probe, where accuracy can be as low as ±0.2 in seawater unless careful calibration to spectrophotometric pH is performed [41]. 

At a low cost and with minimal training, the pHyter can enable people around the world to gain similar insight into the pH trends of their local waters that scientists are able to observe with more expensive and technical instruments. With the pHyter, surveys such as the Duxbury Bay study presented in this article could be frequently conducted by nearly anyone, enabling non-profit organizations, governmental regulators, students, and citizen scientists to survey the pH of their coastlines to inform decisions and policy. The pHyter is also an excellent tool for STEM and OA education programs and can be used to engage and stimulate students and help them learn about local and global environmental issues. The pHyter is one verified and tested tool that can help achieve the vision of affordable, accessible, and accurate pH monitoring devices that allow people around the world to make pH measurements in their local ecosystems.

## Figures and Tables

**Table 1:** (pHyter measurements of Tris CRM pH). Measurement of Tris pH by five individual pHyter instruments at three different temperatures. The average and standard deviation are across the five instruments. The value in red indicates an apparent outlier. Values in parentheses indicate the average and standard deviation without the outlier.

Temperature (C)	Tris pH	pHyter 1	pHyter 2	pHyter 3	pHyter 4	pHyter 5	Average Offset	Standard Deviation
24.74 ± 0.09	8.107 ± 0.003	8.130	8.120	8.120	8.120	8.110	+0.013	±0.007
22.09 ± 0.35	8.190 ± 0.011	8.230	8.220	8.230	8.200	8.190	+0.024	±0.010
27.72 ± 0.19	8.016 ± 0.006	8.030	8.189	8.010	8.013	N/A	+0.046 (+0.002)	±0.086 (0.011)

## Data Availability

Data is available upon request.
